# Plasma Prostaglandin E_2_ Metabolite Levels Predict Type 2 Diabetes Status and One-Year Therapeutic Response Independent of Clinical Markers of Inflammation

**DOI:** 10.3390/metabo12121234

**Published:** 2022-12-08

**Authors:** Rachel J. Fenske, Alicia M. Weeks, Michael Daniels, Randall Nall, Samantha Pabich, Allison L. Brill, Darby C. Peter, Margaret Punt, Elizabeth D. Cox, Dawn Belt Davis, Michelle E. Kimple

**Affiliations:** 1 Research Service, William S. Middleton Memorial VA Hospital, Madison, WI 53705, USA; 2 Department of Nutritional Sciences, University of Wisconsin-Madison, Madison, WI 53706, USA; 3 Department of Clinical Nutrition, UW Health University Hospital, Madison, WI 53705, USA; 4 Department of Medicine, Division of Endocrinology, Diabetes, and Metabolism, University of Wisconsin-Madison, Madison, WI 53706, USA; 5 Department of Pediatrics, University of Wisconsin-Madison, Madison, WI 53792, USA; 6 Department of Cell and Regenerative Biology, University of Wisconsin-Madison, Madison, WI 53792, USA

**Keywords:** type 2 diabetes, prostaglandin E_2_, inflammation, diabetes control, biomarker, HbA1c, plasma metabolites, clinical study

## Abstract

Over half of patients with type 2 diabetes (T2D) are unable to achieve blood glucose targets despite therapeutic compliance, significantly increasing their risk of long-term complications. Discovering ways to identify and properly treat these individuals is a critical problem in the field. The arachidonic acid metabolite, prostaglandin E_2_ (PGE_2_), has shown great promise as a biomarker of β-cell dysfunction in T2D. PGE_2_ synthesis, secretion, and downstream signaling are all upregulated in pancreatic islets isolated from T2D mice and human organ donors. In these islets, preventing β-cell PGE_2_ signaling via a prostaglandin EP3 receptor antagonist significantly improves their glucose-stimulated and hormone-potentiated insulin secretion response. In this clinical cohort study, 167 participants, 35 non-diabetic, and 132 with T2D, were recruited from the University of Wisconsin Hospital and Clinics. At enrollment, a standard set of demographic, biometric, and clinical measurements were performed to quantify obesity status and glucose control. C reactive protein was measured to exclude acute inflammation/illness, and white cell count (WBC), erythrocyte sedimentation rate (ESR), and fasting triglycerides were used as markers of systemic inflammation. Finally, a plasma sample for research was used to determine circulating PGE_2_ metabolite (PGEM) levels. At baseline, PGEM levels were not correlated with WBC and triglycerides, only weakly correlated with ESR, and were the strongest predictor of T2D disease status. One year after enrollment, blood glucose management was assessed by chart review, with a clinically-relevant change in hemoglobin A1c (HbA1c) defined as ≥0.5%. PGEM levels were strongly predictive of therapeutic response, independent of age, obesity, glucose control, and systemic inflammation at enrollment. Our results provide strong support for future research in this area.

## 1. Introduction

Pre-diabetes and diabetes directly affect over 100 million people in the United States. Type 2 diabetes (T2D), which is strongly associated with obesity and inflammation, accounts for 95% of these diagnoses [1]. Current standards of care include diet and lifestyle modifications, oral and injectable anti-diabetic drugs, and insulin. Yet, despite therapeutic compliance, over 50% of T2D patients are unable to achieve blood glucose targets, as defined by a hemoglobin A1C (HbA1c) value of less than 7% [2]. Being able to determine which patients are at risk for therapeutic failure remains a significant problem in the field. A precision (i.e., personalized) medicine approach that recognizes and incorporates the many individual differences noted in practice is a significant focus of current research in the field [3,4,5,6,7,8].

PGE_2_ is an arachidonic acid metabolite, and its formation is catalyzed by a series of enzymes, with cyclooxygenase (COX) 1 or 2 catalyzing the rate-limiting step. COX-2 expression is inducible and its activity and/or expression is significantly elevated in the hyperglycemic, dyslipidemic, and/or inflammatory conditions associated with T2D [9,10,11,12,13,14,15,16,17]. In preclinical work using mouse models of T2D and/or pancreatic islets obtained from human organ donors with T2D, prostaglandin E_2_ (PGE_2_) limits expected insulin secretion in response to both glucose and incretin hormones such as glucagon-like peptide 1 (GLP-1), actively contributing to β-cell dysfunction [9,10,11]. Furthermore, arachidonic acid, its precursors, and/or its metabolites have been shown to be elevated in biofluids (e.g., plasma, serum, urine) from both animals and human subjects with T2D [18,19,20,21,22]. Taken together, these preclinical data provide strong support for pursuing PGE_2_ as a potential biomarker for T2D status and, potentially, therapeutic response.

In this work, we conducted a cross-sectional analysis of adults with T2D to determine if plasma levels of PGE_2_ metabolite (PGEM) correlated with T2D status, comparing PGEM to other established markers of glucose control and inflammation. Finally, baseline PGEM levels were compared to longitudinal glycemic control (as measured by percent change in HbA1c over 1 year), thus providing insight into the relevance of PGE_2_ to T2D therapeutic response.

## 2. Materials and Methods

### 2.1. Study Design, Intake Appointment, and Plasma Sample Collection

Study design and participant recruitment have been previously described [21]. In brief, 132 individuals with T2D and 35 non-diabetic individuals were enrolled between June 2014 and August 2015 at UW Health Hospitals and Clinics (UWHC). Inclusion criteria were ages 18–74, not pregnant or lactating, no anemia or grossly abnormal kidney or liver function tests, no known autoimmune diseases or inflammatory disorders, and no diagnosis of diabetes besides T2D. Exclusion criteria included the history of transplant, chronic steroid use, or the use of COX inhibitors other than low-dose aspirin for cardiovascular health more than twice per week during the past 90 days. Subjects were instructed to fast for 10 h prior to an upcoming diabetes standard-of-care care appointment, where biometric measurements and clinical laboratory tests were performed. Height and weight (to calculate BMI), blood pressure, and pulse were measured, and daily prophylactic low-dose aspirin and omega-3/fish oil supplement use were noted. Current T2D medications were confirmed and recorded. Diabetes standard-of-care laboratory tests, including HgA1c, complete metabolic panel (CMP), and fasting lipid panel, were coordinated with the patient’s provider. Additional clinical laboratory tests performed for research included white blood cell count (WBC), C reactive protein (CRP), and erythrocyte sedimentation rate (ESR). A plasma sample for research was collected in an 8.5 mL BD P800 blood collection tube coated with potassium EDTA and a proprietary mix of protease and esterase inhibitors (BD Biosciences, Franklin Lakes, NJ, USA, cat. No. 366421) for downstream analysis of PGEM levels.

### 2.2. Prostaglandin E Metabolite (PGEM) Assay

Plasma PGE_2_ levels were quantified using a Prostaglandin E Metabolite (PGEM) enzyme-linked immunosorbent assay (ELISA) kit (Cayman Chemical Company, Ann Arbor, MI, USA, cat. No. 514531), which converts 13,14-dihydro-15-keto PGE_2_ and 13,14-dihydro-15-keto PGE_2_ to a single, stable derivative. The assay was conducted according to the manufacturer’s protocol, as previously described [21]. Briefly, after samples were purified by acetone precipitation, they were dried under a nitrogen stream. Samples were then resuspended in ELISA buffer and derivatized overnight. A 1:5 sample dilution was assayed in duplicate.

### 2.3. Statistical Analysis

Logistic regression analysis was used to determine the relationship between plasma PGEM levels and T2D status. SAS software was used with a Probit procedure for this analysis (SAS/STAT, Cary, NC, USA). All other statistical analyses were performed using GraphPad Prism version 9 (GraphPad Software, San Diego, CA, USA), and data were compared by one- or two-way analysis of variance or Student *t*-test as appropriate, as described in the figure legends. A *p*-value < 0.05 was considered statistically significant.

## 3. Results

### 3.1. Plasma PGEM Is Increased Specifically in Subjects with T2D

Demographic information for the non-diabetic (ND) control group (*n* = 35) and T2D group (*n* = 132) are listed in Table 1. Most subjects were white/non-Hispanic, and approximately equal numbers of male and female subjects were represented (Table 1).

**Figure 1:**
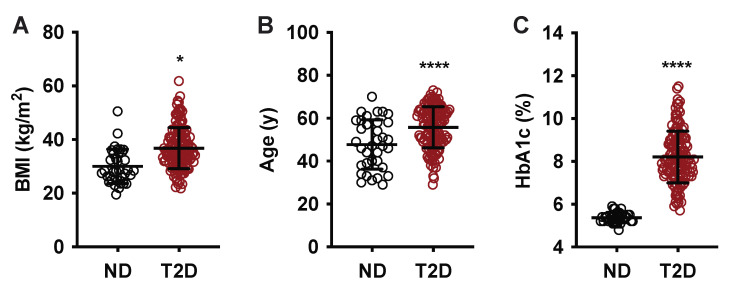
Baseline BMI, age, and HbA1c of ND and T2D subjects at enrollment. (**A**) Body mass index (BMI); (**B**) age in years; (**C**) glycated hemoglobin (HbA1c) for non-diabetic group (black circle) and T2D group (red circle). Data are presented as mean ± standard deviation. *, *p* < 0.05; ****, *p* < 0.0001. Black.

Subject age range was similar for both groups (29–70 years, ND vs. 29–73 years, T2D), although the means were statistically different (47.8 ± 11.5, ND vs. 55.8 ± 9.6 T2D; *p* < 0.0001) (Table 1 and Figure 1A). Like age, the BMI range was similar between groups (19.48–50.51, ND vs. 21.79–61.85), although the difference in mean BMI was statistically significant (29.89 ± 6.52, ND vs. 36.79 ± 7.72, T2D; *p* < 0.0001) (Table 1 and Figure 1B). The mean HbA1c for the T2D group was significantly higher than that of the ND group, as expected (5.4 ± 0.2, ND vs. 8.2 ± 1.2, T2D; *p* < 0.0001) (Table 1 and Figure 1C).

### 3.2. In T2D Subjects, Plasma PGEM Is Only Weakly Correlated with Systemic Inflammation

**Figure 2:**
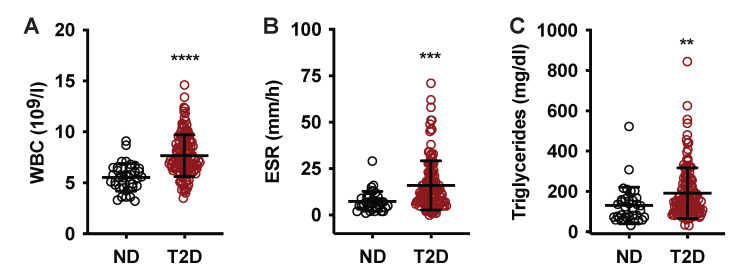
Relationship between T2D diagnosis and markers of inflammation. (**A**) White blood cell count (WBC); (**B**) erythrocyte sedimentation rate (ESR); (**C**) plasma triglycerides for non-diabetic group (black circle) and T2D group (red circle). Data are presented as mean ± standard deviation. **, *p* < 0.01; ***, *p* < 0.001; ****, *p* < 0.0001.

WBC and ESR are clinical tests for systemic inflammation that have both been validated as markers of disease risk and progression in pre-diabetic and T2D populations [23,24,25,26,27,28,29,30,31,32,33,34,35]. No T2D subjects (0%) and 8 T2D subjects (6.1%) had elevated WBC (Table 1), and mean WBC was statistically higher in T2D subjects as compared to ND (Table 1 and Figure 2A). One ND subject (2.9%) and 27 T2D subjects (20.5%) had elevated ESR for age and sex (Table 1), and the mean ESR was also significantly higher in T2D subjects (16 mm/h vs. 7.5 mm/h, respectively) (Table 1 and Figure 2B). Elevated triglycerides are known to be associated with inflammation, metabolic syndrome, and T2D status and risk [36,37,38], and triglyceride levels were elevated in T2D subjects as compared to ND (Table 1 and Figure 2C). No subjects had CRP levels over 10, the baseline for moderate inflammation, confirming the absence of acute infection or injury (data not shown).

### 3.3. Plasma PGEM Is a Strong Predictor of T2D Disease Status

**Figure 3:**
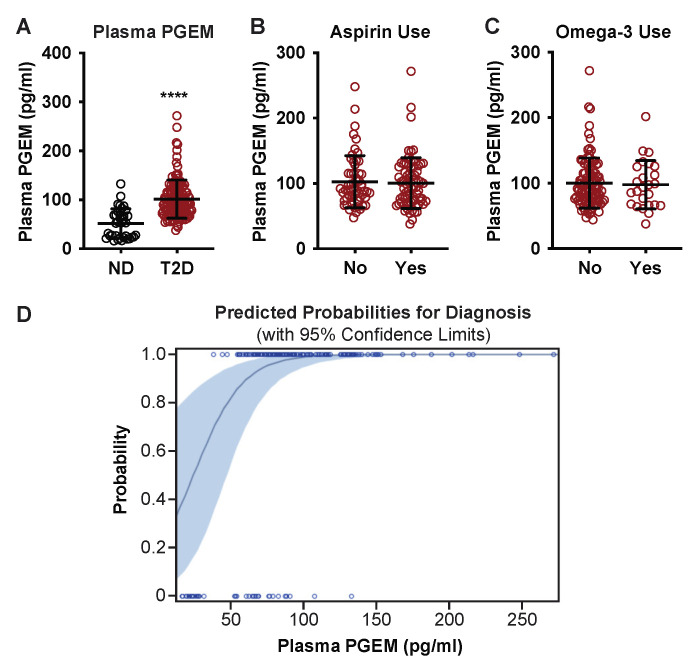
Plasma PGEM is elevated in T2D subjects and is a strong predictor of T2D diagnosis. (**A**) Plasma PGEM levels of ND and T2D subjects at enrollment; (**B**) plasma PGEM levels of T2D subjects based on daily prophylactic aspirin use; (**C**) plasma PGEM levels at enrollment of T2D subjects based on daily omega-3/fish oil supplement use. In (**A**–**C**), data are presented as mean ± standard deviation. ****, *p* < 0.0001. (**D**) Predictive probability of plasma PGEM of T2D diagnosis generated by the SAS Probit process.

Plasma PGEM levels were, on average, two-fold higher in the T2D group as compared to ND controls (51.6 ± 30.4, ND vs. 101.5 ± 39.1, T2D; *p* < 0.0001) (Table 1 and Figure 3A). There were no statistically significant correlations between plasma PGEM and BMI, HbA1c, WBC, and triglycerides, and only a weak correlation with ESR (Table 1). The rate-limiting step in PGE_2_ production is catalyzed by cyclooxygenase (COX) enzymes, and aspirin is a COX-1 inhibitor [39]. We found no difference in mean PGEM levels between those in the T2D group who reported low-dose daily aspirin use (*n* = 54) and those who did not (*n* = 71) (Figure 3B). Eicosapentaenoic acid (EPA) is an omega-3 polyunsaturated fatty acid that competes with arachidonic acid for the same site in plasma membrane phospholipids [10]. We found no difference in mean PGEM between those in the T2D group who reported omega-3/fish oil supplement use (*n* = 37) and those who did not (*n* = 95) (Figure 3C). A logistic regression analysis including age, sex, BMI, WBC, triglycerides, aspirin use, and PGEM revealed PGEM as the strongest predictor of T2D diagnosis (*p* < 0.0001) (Figure 3D and Table 2) (ESR was not included in this analysis as it was strongly associated with WBC (*p* < 0.0001; R^2^ = 0.12).

### 3.4. T2D Patients with High Plasma PGEM Levels Have Significantly Worse Blood Glucose Control One-Year Post-Enrollment

T2D subjects were assessed by chart review one year following the study enrollment, and their percent change in HbA1c was calculated. Seven subjects were lost to follow-up, and three additional subjects whose HbA1c increased more than 4% were excluded due to non-compliance. The final analysis included 45 subjects with a clinically significant reduction in HbA1c (≥0.5%) one-year post-enrollment and 77 without (*n* = 122).

**Figure 4:**
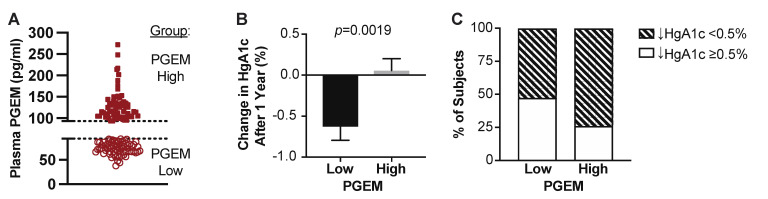
Subjects with high plasma PGEM have worse T2D therapeutic control one-year after enrollment. (**A**) Plasma PGEM levels of T2D subjects at enrollment below and above the median of 92.96 pg/mL; (**B**) percent change in HbA1c for the subjects shown in (**A**). (**C**) Percent of subjects in “low” and “high” PGEM groups with or without a clinically meaningful reduction in HbA1c of ≥ 0.5% (white and hatched bars, respectively).

As there is no clinical threshold for plasma PGEM, the median PGEM level from all 132 T2D patients (92.96 pg/mL) was used to classify T2D subjects in either a “low” or “high” PGEM group for follow-up analyses (Figure 4A). On average, T2D subjects with low plasma PGEM exhibited a 0.6% decrease in HbA1c: a statistically significant difference from those in the high PGEM group, where no change in mean HbA1c was observed (*p* = 0.0019) (Figure 4B). In total, 47.5% of T2D patients with low plasma PGEM levels achieved a clinically significant reduction in HbA1c (≥0.5%) over 1 year (Figure 4C). Conversely, only 25.8% of T2D patients with high plasma PGEM were able to achieve a clinically significant reduction in HbA1c over 1 year (Figure 4C).

## 4. Discussion

In this study, we demonstrate that plasma PGEM shows promise as a circulating biomarker to assess the risk of T2D diagnosis and the efficacy of blood glucose management in individuals with T2D. Plasma levels of a stable metabolite of PGE_2_ were significantly higher in individuals living with T2D when compared to a control group. This finding is consistent with recent work from our group and others using small numbers of biosamples from obese, ND, and T2D subjects [18,20,21,22]. These results with a larger clinical cohort both validate the previous findings, as well as reveal PGEM as a strong predictor of T2D disease status: even more so than validated clinical tests of systemic inflammation. Finally, for the first time, we discovered plasma PGEM was a strong predictor of T2D therapeutic response over the following year. Our findings provide strong evidence for further investigations into the role of PGE_2_ metabolites in diabetes pathogenesis and treatment response.

The expression and/or activity of COX enzymes, which catalyze the rate-limiting step in PGE_2_ production, are significantly upregulated by pro-inflammatory cytokines [9,10,11,12,13,14,15,16,17,18,40,41,42,43,44,45]. T2D is a pathophysiological state strongly associated with adipose meta-inflammation and insulin resistance [46,47,48,49], with a number of validated clinical tests of systemic inflammation correlating with T2D disease risk and status [23,24,25,26,27,28,29,30,31,32,33,34,35]. Outside of its canonical role as an inflammatory signaling molecule, though, PGE_2_ has been shown to play an important role in the β-cell’s function and survival [9,10,15,16,17,21,50,51]. These findings suggest PGE_2_ signaling may contribute to all three of the primary underlying defects in the progression to and development of T2D—insulin resistance, elevated fasting glucose, and glucose intolerance—and is worthy of future study.

The gut microbiome is well-known to be associated with obesity and T2D [52,53,54]. The composition of the gut microbiome strongly influences circulating metabolites and has also been shown to influence incretin sensitivity in pre-diabetes and T2D [55,56,57]. In previous work using a mouse model of T2D, we found the composition of the gut microbiome was associated with systemic metabolomic changes, including elevated arachidonic acid, that correlated with islet-level PGE_2_ production and responsiveness to a PGE_2_ receptor agonist [14]. While outside of the scope of this study, future work studying the relationship between the gut microbiome and plasma PGEM levels with T2D outcomes is warranted.

While obesity is a driver of T2D pathology, and changes in plasma PGEM could indicate glucolipotoxic metabolic and inflammatory dysfunction, we demonstrated no biologically relevant correlations to other biomarkers of obesity, inflammation, or insulin resistance, including BMI, WBC, ESR, and triglyceride levels (Table 1, Figure 1 and Figure 2). Logistic regression analysis of the data set, including age, sex, BMI, WBC, triglycerides, and aspirin use indicated the most significant predictor of T2D status was plasma PGEM, and subjects having plasma PGEM levels greater than 101.5 pg/mL had a 99% probability of a T2D diagnosis (Figure 3D and Table 2).

Currently, recommendations by the American Diabetes Association (ADA) suggest that testing to assess risk for future diabetes in asymptomatic people should be considered in adults of any age who are overweight or obese and have one or more additional risk factors, including physical inactivity, first-degree relative with diabetes, women with gestational diabetes mellitus, hypertension, women with polycystic ovary syndrome, history of cardiovascular disease, and others [58]. However, current diagnostic tests are imperfect and prone to misclassification errors. HbA1c has not been validated for all populations [59] and can be confounded by structural variants in the hemoglobin molecule or alterations in red blood cell turnover. The use of plasma PGEM as an additional measure of T2D status could act as a secondary means of quantifying T2D risk assessment. The inclusion of plasma PGEM in this list of risk factors may capture high-risk individuals who may be otherwise go undiagnosed.

Historically, clinical values including HbA1c, intact proinsulin, adiponectin, and high sensitivity C-reactive protein have been suggested as biomarkers of β-cell failure and insulin resistance, although their overall useability is limited, as both specificity and context need to be considered [60]. Individualized treatments based on the precision understanding of an individual’s disease process have garnered much enthusiasm. Ahlqvist et al., used cluster analysis to define five subgroups of individuals based on their diabetes characteristics and risk for developing diabetic kidney disease using six parameters (BMI, HbA1c, glutamic acid decarboxylase antibodies, and homeostatic model assessment of insulin resistance (HOMA-IR) and insulin secretion (HOMA-B) [61]. The results of this study suggested the need to identify additional biomarkers to improve sensitivity and precision in stratifying individuals with pre-diabetes and T2D. Recently, dihydroceramides have been shown to act as a potential biomarker for T2D [7]. In addition to predicting T2D disease status, PGEM is one of a few putative biomarkers that may also provide functional insight into β-cell health relevant to the present therapeutic landscape. Specifically, the expression of PGE_2_ synthetic and signaling enzymes is higher in pancreatic islets isolated from T2D mice and human organ donors than in non-diabetic controls [9,10,14,21,41,51,62,63,64], resulting in significantly elevated PGE_2_ release [9,10]. In the β-cell, PGE_2_ binds to the G_z_-coupled prostaglandin E_2_ EP3 receptor (EP3) [13,40,65,66,67], which, when activated, limits insulin secretion in response to glucose and glucagon-like peptide 1 receptor (GLP1R) agonists: a mechanism that actively contributes to the β-cell dysfunction of the disease [9,67]. GLP1-RAs are currently in wide use as first- or second-line T2D therapeutics, yet, despite the popularity of these drugs in the clinic, they do not have the same efficacy in all patients. With the known inhibitory effect of PGE_2_ receptor antagonists on the efficacy of GLP1-RAs in preclinical models, this finding may be of great importance in clinical decision-making. One limitation is that we did not directly measure β-cell function by methods such as quantifying stimulated C-peptide levels; therefore, this possibility remains only theoretical. Another limitation of the current observational study is it was neither adequately powered nor designed to assess the specific impact of PGEM levels on GLP1-RA efficacy. Future work must include trials of drug-naïve patients with T2D randomized to different classes based on plasma PGEM levels to determine if these could be used to help providers choose the drug that will work best for each patient.

We acknowledge several other limitations of this study. The population in the UWHC catchment area is primarily white/non-Hispanic; therefore, our results may not be representative of more diverse populations. This is an important limitation, as recent studies have found the appropriateness of common biomarkers of T2D risks differ, based on an individual’s racial and ethnic background [68]. Second, as this was an observational study with the primary outcome being plasma PGEM, we did not control for time since T2D diagnosis or current T2D therapeutics. The ongoing management of the subjects’ T2D during the 1 year follow-up period was not influenced in any way by this research study and, therefore, was based on “real-world” standard clinical care. Third, diet quantity/composition and physical activity can impact diabetes control, and in the current study, we did not have participants keep diet or exercise logs to quantify this potential confounder. Finally, as plasma PGEM in a T2D clinical cohort has not previously been studied, it will be necessary to identify and validate an appropriate clinical threshold if it is to be used as a biomarker for T2D therapeutic response. These additional considerations are important to note but fall outside the scope of this study.

## 5. Conclusions

In this clinical cohort study, we find plasma PGEM levels are an excellent predictor of T2D status and one-year therapeutic response, independent of known markers of inflammation, obesity, and T2D disease control. These findings were surprising, as hyperglycemia, dyslipidemia, and pro-inflammatory cytokines have all been shown to upregulate enzymes in the PGE_2_ production pathway. Our results provide strong support for future research into plasma PGEM as an independent biomarker for T2D status and long-term disease control.

## Figures and Tables

**Table 1:** Demographic and clinical parameters of the patient cohort. Unless otherwise indicated, data are presented as mean ± standard deviation. BMI, body mass index; HbA1c, glycated hemoglobin; WBC, white blood cell count; ESR, erythrocyte sedimentation rate; PGEM, PGE_2_ metabolite.

	Groups			ND vs. T2D	Linear Regression vs. PGEM (T2D Only)	
Baseline Demographics						
	All	Non-Diabetic	T2D	*p*-Value	*p*-Value	R^2^
Subjects (*n*)	167	35	132	-	-	-
Male (*n*; %)	85; 51%	13; 37%	72; 55%	-	-	-
Female (*n*; %)	82; 49%	22; 63%	60; 45%	-	-	-
Race/Ethnicity = White/Non-Hispanic (*n*; %)	153; 92%	32; 91%	121; 92%	-	-	-
Age (years ± SD; range)	54.1 ± 10.5; 29–73	47.8 ± 11.5; 29–70	55.8 ± 9.6; 29–73	<0.0001	0.84	0.0003
Baseline Biometric and Laboratory Parameters						
BMI (kg/m^2^ ± SD; range)	35.2 ± 8.3; 19.5–61.9	29.9 ± 6.5; 19.5–50.5	36.8 ± 7.7; 21.8–61.9	<0.0001	0.79	0.0005
HbA1c (% ± SD; range)	7.6 ± 1.6	5.4 ± 0.2	8.2 ± 1.2	<0.0001	0.79	0.0006
WBC (10^9^/L ± SD)	7.2 ± 2.1	5.5 ± 1.4	7.7 ± 2.0	<0.0001	0.25	0.01
ESR, mm/h (mm/h ± SD)	14.2 ± 12.5	7.5 ± 5.4	16.0 ± 13.2	0.0003	0.035	0.034
Triglycerides (mg/dL ± SD)	179.6 ± 121.3	131.2 ± 90.8	191.1 ± 125.5	0.009	0.79	0.007
Plasma PGEM, (pg/mL ± SD)	91.0 ± 42.5	51.6 ± 30.4	101.5 ± 39.1	<0.0001	-	-

**Table 2:** Logistic regression analysis of PGEM and other baseline parameters reveals plasma PGEM as the strongest predictor of T2D diagnosis. The SAS Probit procedure was used.

	Analysis of Maximum Likelihood Parameter Estimates					
Parameter	DF	Estimate	Std. Error	95% CI	ChiSq	Pr > ChiSq
Intercept	1	−15.8799	3.3533	−22.4522 to −9.3076	22.43	<0.0001
PGEM	1	0.0593	0.0141	−0.0317 to 0.0869	17.70	<0.0001
Age	1	0.1001	0.0375	0.0265 to 0.1737	7.71	0.0076
BMI	1	0.0765	0.0449	−0.0166 to 0.1746	2.90	0.0886
WBC	1	0.7225	0.2341	0.2635 to 1.1814	9.52	0.0020
Triglycerides	1	0.0064	0.0042	−0.0019 to 0.0147	2.25	0.1332
Sex	1	−0.9199	0.6979	−2.2878 to 0.4480	1.74	0.1875
Aspirin use	1	−23.689	180, 132.4	−353,077 to 353,023	0.00	0.9999

## Data Availability

Not applicable.
